# Neurological Complications of Infective Endocarditis

**DOI:** 10.1590/0037-8682-0319-2025

**Published:** 2025-09-29

**Authors:** Lucas Yunes Cominatto Barbosa, Laisson de Moura Feitoza, Luiza Maretti Scomparin, Lucieni Oliveira Conterno, Fabiano Reis

**Affiliations:** 1 Universidade Estadual de Campinas, Departamento de Radiologia e Oncologia, Campinas, SP, Brasil.; 2 Universidade Estadual de Campinas, Departamento de Oftalmologia e Otorrinolaringologia, Campinas, SP, Brasil.; 3 Universidade Estadual de Campinas, Departamento de Clínica Médica, Campinas, SP, Brasil.

A 40-year-old man presented with painless, sudden-onset visual loss. He also reported daily fever, morning polyarthralgia, and a 20-kg weight loss.

His medical history included recent dental procedures and chronic alcohol use. Physical examination revealed that the patient was malnourished and had poor dental hygiene. Cardiac auscultation revealed a holosystolic murmur in the mitral area. 

These findings raised clinical suspicion of infective endocarditis (IE) according to the modified Duke criteria.

Transthoracic echocardiography revealed a mobile vegetation on the mitral valve. The diagnosis of IE was confirmed when *S. sanguinis* was detected in blood culture. The patient received targeted antibiotic therapy with ceftriaxone (2 g/day) and gentamicin (3 mg/kg/day), and subsequently underwent dental treatment.

On the thirteenth day of hospitalization, the patient developed a sudden severe headache accompanied by a transient loss of consciousness. Head computed tomography (CT) was performed (Figure 1A-C).

**FIGURE 1:**
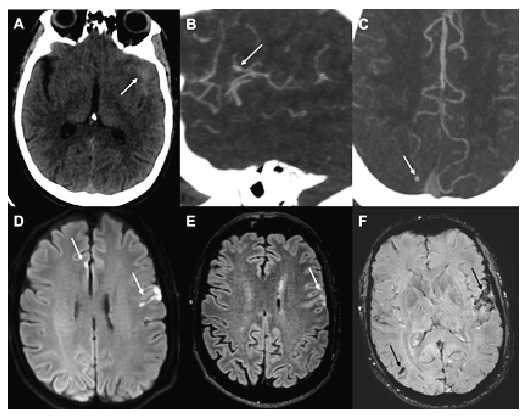
Head computed tomography (CT) showing a thin subarachnoid hemorrhage in the left Sylvian fissure (**arrow in A**). CT angiography demonstrating a small fusiform dilation in an opercular branch of the left middle cerebral artery (**arrow in B**) and a punctiform enhancement in the right superior parietal lobule (**arrow in C**), both of which were suggestive of mycotic aneurysms. Brain diffusion-weighted magnetic resonance imaging and FLAIR revealed multiple cortical infarcts (**white arrows in D and E**), consistent with embolic stroke. Susceptibility-weighted Imaging also showed microbleeds in the left frontal and right parietal sulci (**arrows in F**).

Brain magnetic resonance imaging (MRI) (Figure 1D-F) and digital subtraction angiography (Figure 2A,B) were also performed. 

A few days after this neurological diagnosis, a new head CT was performed (Figure 2C,D). 

**FIGURE 2:**
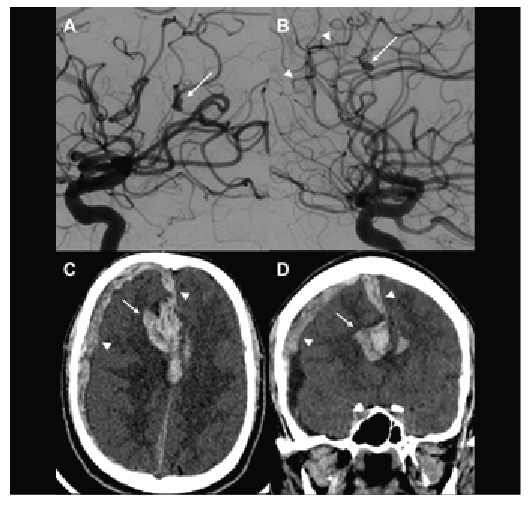
Digital subtraction angiography confirmed a fusiform aneurysm in an opercular branch of the left middle cerebral artery (**arrow in A**) and showed another fusiform aneurysm in the right pericallosal artery (**arrow in B**), consistent with mycotic aneurysms. Tiny irregularities were also observed in the frontal branches of the right anterior cerebral artery (**arrowheads in B**) associated with inflammatory vasculopathy. Axial and coronal head computed tomography 28 days after admission showed right frontal intraparenchymal hemorrhage (arrows) and right frontoparietal and inter-hemispheric acute subdural hematoma (white arrowheads). Uncal herniation and a downward displacement of the cerebellar tonsils (not shown) were also observed.

Neurological complications occur in approximately 25% of patients with IE. The most common manifestations include ischemic stroke, intracranial hemorrhage, cerebral abscess, and meningitis^1^ ^,^ ^2^. Mycotic aneurysms, although less common, pose a serious risk because of the high likelihood of rupture^2^ ^,^ ^3^. Although rare, cerebral aneurysms can lead to subdural hematoma, as observed in this case^4^. Recognizing these atypical presentations highlights the importance of prompt diagnosis, appropriate imaging, and timely treatment of underlying infections.

## References

[1] de Amorim JC, Torricelli AK, Frittoli RB, Lapa AT, Dertkigil SSJ, Reis F (2018). Mimickers of neuropsychiatric manifestations in systemic lupus erythematosus. Best Pract Res Clin Rheumatol.

[2] Sotero FD, Rosário M, Fonseca AC, Ferro JM (2019). Neurological Complications of Infective Endocarditis. Curr Neurol Neurosci Rep.

[3] Lee WK, Mossop PJ, Little AF, Fitt GJ, Vrazas JI, Hoang JK (2008). Infected (mycotic) aneurysms: spectrum of imaging appearances and management. Radiographics.

[4] Caton MT, Wiggins WF, Nuñez D (2019). Non-traumatic subdural hemorrhage: beware of ruptured intracranial aneurysm. Emerg Radiol.

